# O.4.2-4 The influence of organized sport participation on health-related fitness development during adolescence

**DOI:** 10.1093/eurpub/ckad133.179

**Published:** 2023-09-11

**Authors:** Iiris Kolunsarka, David Stodden, Arto Gråstèn, Mikko Huhtiniemi, Timo Jaakkola

**Affiliations:** Faculty of Sport and Health Sciences, University of Jyväskylä, Finland; College of Education, University of South Carolina, Columbia, SC, USA; Faculty of Sport and Health Sciences, University of Jyväskylä, Finland; College of Education, United Arab Emirates University, Al Ain, United Arab Emirates; iiankolu@jyu.fi; Faculty of Sport and Health Sciences, University of Jyväskylä, Finland; Faculty of Sport and Health Sciences, University of Jyväskylä, Finland

## Abstract

**Purpose:**

Organized sport participation plays particularly big role in promoting adolescents’ physical activity and health-related fitness. However, it remains to be understood how current, past, or complete non-participation in organized sports affect adolescents’ individual trajectories of cardiorespiratory and muscular fitness and whether adolescents benefit from their past participation in organized sports in terms of fitness. Thus, the aim of this research was to study the development of cardiorespiratory and muscular fitness during adolescence in those 1) who continued or started organized sport participation, 2) who dropped out, and 3) who never participated or dropped out before adolescence.

**Methods:**

In this five-year longitudinal study, which was carried out between 2017-2021, self-reported sport participation data were collected annually, and cardiorespiratory and muscular fitness measurements were collected every other year from 963 participants (Mage = 11.25 ± 0.31). Multi-group parallel latent growth curve model was used to examine initial levels and slopes (rate of change) of cardiorespiratory and muscular fitness simultaneously in each of three sport participation group: 1) continuous, 2) dropout, and 3) non-part.

**Results:**

Continuous group had significantly higher initial levels of cardiorespiratory and muscular fitness compared to other two groups. Moreover, both continuous and non-part groups had positive slopes of cardiorespiratory and muscular fitness. Although dropout group had significantly higher initial levels of cardiorespiratory and muscular fitness compared to non-part group, the slopes were smaller or not significant. Thus, at the last time point, there were no longer significant differences between these two groups in levels of cardiorespiratory and muscular fitness.

**Conclusions:**

Continued participation in organized sports promotes adolescents’ health-related fitness. However, as adolescents dropout these health-related fitness improvements seem to be lost. Therefore, it is necessary to consider what could be done so that adolescents who dropout or never participated in organized sports would have equal opportunities to promote their health-related fitness. One reason for dropout is that organized sports tend to get more competitive, time-consuming, and expensive in adolescence. Thus, solution could be low-cost and low-threshold recreational or school-related physical activities that could improve or maintain health-related fitness during adolescence.

**Funding Source:**

Finnish Ministry of Education and Culture

